# Horizontally Acquired Homologs of Xenogeneic Silencers: Modulators of Gene Expression Encoded by Plasmids, Phages and Genomic Islands

**DOI:** 10.3390/genes11020142

**Published:** 2020-01-29

**Authors:** Alejandro Piña-Iturbe, Isidora D. Suazo, Guillermo Hoppe-Elsholz, Diego Ulloa-Allendes, Pablo A. González, Alexis M. Kalergis, Susan M. Bueno

**Affiliations:** 1 Millennium Institute on Immunology and Immunotherapy, Departamento de Genética Molecular y Microbiología, Facultad de Ciencias Biológicas, Pontificia Universidad Católica de Chile, 8331010 Santiago, Chile; llpina@uc.cl (A.P.-I.); idsuazo@uc.cl (I.D.S.); gahoppe@uc.cl (G.H.-E.); dvulloa@uc.cl (D.U.-A.); pagonzalez@bio.puc.cl (P.A.G.); akalergis@bio.puc.cl (A.M.K.); 2 Departamento de Endocrinología, Facultad de Medicina, Pontificia Universidad Católica de Chile, 8331010 Santiago, Chile

**Keywords:** genomic islands, xenogeneic silencers, horizontal gene transfer, transcriptional network, H-NS, MvaT, Lsr2, Rok

## Abstract

Acquisition of mobile elements by horizontal gene transfer can play a major role in bacterial adaptation and genome evolution by providing traits that contribute to bacterial fitness. However, gaining foreign DNA can also impose significant fitness costs to the host bacteria and can even produce detrimental effects. The efficiency of horizontal acquisition of DNA is thought to be improved by the activity of xenogeneic silencers. These molecules are a functionally related group of proteins that possess affinity for the acquired DNA. Binding of xenogeneic silencers suppresses the otherwise uncontrolled expression of genes from the newly acquired nucleic acid, facilitating their integration to the bacterial regulatory networks. Even when the genes encoding for xenogeneic silencers are part of the core genome, homologs encoded by horizontally acquired elements have also been identified and studied. In this article, we discuss the current knowledge about horizontally acquired xenogeneic silencer homologs, focusing on those encoded by genomic islands, highlighting their distribution and the major traits that allow these proteins to become part of the host regulatory networks.

## 1. Introduction

Horizontal gene transfer (HGT) refers to the natural process of transmission of genetic material to an organism in addition to the inheritance of genes from parents to offspring [1]. The availability of an increasing number of sequenced bacterial genomes has revealed the great extent of HGT in these organisms, which highlights the major role played by this process in bacterial adaptation to the environment and to the hosts in which they thrive [2]. Several functions, such as those related to niche colonization, symbiotic relationships, catabolism of new nutrients, antimicrobial resistance, and pathogenesis, can be acquired through the DNA gained by HGT [3,4,5]. However, acquisition of some genetic elements can impose fitness costs onto the recipient bacteria [6,7] likely as a result of the additional DNA that requires replication and repair, in addition to the eventual uncontrolled expression that the newly acquired genes might undergo [8,9,10]. In the past decades, a group of proteins denominated xenogeneic silencers (XSs) was shown to target the horizontally acquired DNA, repressing the expression and contributing to the integration of the horizontally acquired genes into the host transcriptional network [11,12,13,14]. 

While XSs are encoded by the core genome [12,15,16], homologs encoded by horizontally acquired DNA have also been discovered and characterized [17]. In recent years, new findings have shed light on the role played by these XS homologs and several more have been identified in numerous mobile elements, which highlights the extent of their presence. In this article, the current knowledge about the horizontally acquired XS homologs is discussed. After a brief introduction to the main features of the four families of XS proteins, we describe the horizontally acquired homologs and their distribution among bacteria. We also summarize the evidence of the interaction between the horizontally acquired homologs and the core XSs, highlighting the impact produced on the bacterial transcriptional networks. Furthermore, assisted by bioinformatics, we present a more in-depth view about the XS homologs encoded by genomic islands, showing that these proteins are encoded by highly AT-rich islands in *Enterobacteriaceae* and that genes encoding homologs from different XS families are also present in genomic islands. Together, the literature indicates that the horizontally acquired XSs play important roles outside their encoding elements, mostly as a result of the interaction with the core XSs.

## 2. Xenogeneic Silencers

### 2.1. The H-NS, MvaT, Lsr2 and Rok Families of Xenogeneic Silencers

Xenogeneic silencers are nucleoid-associated proteins (NAPs), a diverse group of proteins involved in the condensation of the bacterial chromosome, bending and bridging the DNA to organize it in both micro and macro domains that can easily fit inside a bacterial cell [18,19]. These proteins are also involved in the regulation of replication and transcription, as well as in the reorganization of the nucleoid to provide accessibility to both the DNA- and RNA-polymerases [20,21,22,23]. Four families of XS proteins have been identified so far, defined by sequence and structure. In Gram-negative bacteria, the H-NS and MvaT families are present among several species of Alpha, Beta, and Gammaproteobacteria, with the second present only in Pseudomonadales (Gammaproteobacteria). In Gram-positive bacteria, the Lsr2 and Rok families can be found in Actinobacteria such as *Mycobacterium* spp. and *Bacillus* spp., respectively [24,25,26]. Despite their low sequence similarity, these four families of NAPs share some features that are responsible for their ability to target and bind horizontally acquired DNA, as well as to interact with themselves or with homologs, which leads to gene expression silencing [24].

### 2.2. Binding to AT-Rich DNA and Oligomerization are Key Features for XS Function

Recognition of AT-rich DNA (including core promoters and horizontally acquired DNA) and formation of high-order complexes allow H-NS, MvaT, Lsr2, and Rok to act as global transcriptional regulators of hundreds of genes in their respective bacterial hosts, mainly as repressors of gene expression. For this reason, XSs are involved in the regulation of several key functions, such as replication, transcription, translation, chemotaxis, biofilm formation, modulation of diverse biosynthetic pathways, stress responses and virulence, among others [25,27,28,29]. 

Xenogeneic silencers bind non-specifically to DNA but prefer AT-rich regions, a feature often found in horizontally acquired elements [24,30,31,32,33,34]. For years, the basis of this preference remained elusive until nuclear magnetic resonance studies examined the interaction between the *Salmonella* H-NS_Ctd_ and *Burkholderia* Bv3F_Ctd_ (an H-NS homolog) with DNA. These assays showed that a conserved loop in the C-terminal DNA-binding domain contains an AT-hook-like structure defined by the Q/RGR motif that enters the minor groove [35]. AT-rich DNA has a narrower and deeper minor groove that probably provides favorable electrostatic interactions with the Q/R and R residues, especially in sequences that contain contiguous T and A nucleotides (TpA step), which provide the optimal minor groove narrowness and increased DNA flexibility for H-NS binding [35]. The TpA step can be found in many -10 elements of core promoters bound by RNA polymerase, which account for the H-NS capacity to silence gene expression [36,37]. While the AT-hook-like motif is also present in Lsr2 [35], the MvaT and Rok proteins use different mechanisms for binding to DNA. In MvaT, the amino acid side chains that enter the minor groove come from two different loops of the protein, forming an “AT-pincer” [38]. In Rok, a winged-helix domain binds the DNA along the minor groove [26]. As for H-NS, the DNA-binding affinity of the other XSs is favored by the TpA step [26,36,38], except for Lsr2, which is insensitive to its presence [38]. Despite these differences, the DNA-binding domain of the four xenogeneic silencers is always found in their C-terminal domains.

Formation of dimers and oligomers enable the cooperative binding of XSs to DNA and the formation of bridges within or between different regions of the chromosome. Both the dimerization and oligomerization domains of H-NS, MvaT, and Lsr2 are in the N-terminal region. In these proteins the dimerization/oligomerization results from the interaction of four α-helices in H-NS, two α-helices in the MvaT-homolog TurB, and one antiparallel β-sheet and one α-helix in Lsr2 [39,40,41]. Antiparallel homodimers and heterodimers, as well as higher-order interactions, can be formed as a result of oligomerization, which is important for nucleoid compaction and modulation of gene expression [16,42,43]. Less is known about the oligomerization of Rok, but recent data show that it can form oligomers through the N-terminal domain [26]. It is important to mention that truncated versions of the XS proteins that maintain the N-terminal dimerization domain can exert a dominant-negative effect on the other XS homologs present in the bacterial cell. For instance, in the presence of a null hns mutation, the H-NS paralog StpA can function as a backup that represses genes originally targeted by H-NS [44]; however, if the truncated version of H-NS is present, it forms dimers with StpA, disrupting its function as a result of the absence of the H-NS binding domain [45].

Changes in temperature, pH, or growth phase can alleviate the repression exerted by the XSs on AT-rich genes, enabling expression control in response to environmental stimuli [46], a characteristic that provides a mechanism to integrate horizontally acquired genes into the host transcriptional network. For instance, AT-rich mobile elements can be bound by XSs to repress and avoid deleterious effects resulting from uncontrolled gene expression [24], then the silencing could be relieved in response to particular environmental stimuli in which the functions encoded by the newly acquired genes result beneficial [47,48]. Interestingly, the same features that enable xenogeneic silencers to regulate expression of foreign genes also enable their horizontally acquired homologs to interact with the host transcriptional network (see Section 3). 

## 3. Horizontally Acquired Homologs of Xenogeneic Silencers

Several genes encoding members of the four families of XSs have been identified in hundreds of different horizontally acquired elements, specifically in plasmids, bacteriophages, and genomic islands, which highlights their widespread distribution (Table 1) [49,50,51].

### 3.1. Xenogeneic Silencer Homologs Encoded by Plasmids

The most extensively studied plasmid-encoded homologs are Sfh, Pmr, and Acr2. The H-NS family protein Sfh is encoded on the R27-like plasmids associated with antibiotic resistance in strains of *Salmonella* serovars Typhimurium and Typhi, and on the 99.7% identical plasmid pSf-R27 of *Shigella flexneri* serotype 2a strain 2457T, which lacks the antibiotic resistance genes [58,59,60]. Pmr is an MvaT homolog encoded on the pCAR1 plasmid, which confers carbazole-degrading capacity to *Pseudomonas putida* KT2440 [11,55,64]. Acr2 is an H-NS family protein that acts as a negative regulator of conjugative transfer of plasmids belonging to the A/C incompatibility group, which are responsible for the spreading of antibiotic resistance in several species of Gammaproteobacteria [49,56,65,66]. Acquisition of the plasmids that encode these XS homologs produces transcriptional alterations in the recipient bacteria; however, different changes in the expression of chromosomal and plasmid-encoded genes take place upon acquisition of plasmid variants lacking the corresponding XS-encoding gene, indicating a role of the plasmid-encoded homologs in regulation of gene expression both on and off of their mobile element (see below).

Introduction of the pSf-R27 plasmid in *Salmonella* ser. Typhimurium SL1344 results in altered transcription of several genes related to different functional categories. However, when the Δ*sfh* variant was introduced, an increase in the expression level and the number of gene categories affected was observed along with changes in motility and virulence, which resembled those caused by a dominant-negative *hns* allele [60]. Similarly, the acquisition of the plasmid pCAR1 altered the log-phase transcriptional profile of *P. putida* KT2440, modifying the expression of 112 genes, in which both up- and down-regulation were observed [55]. Interestingly, disruption of *pmr* on pCAR1 altered the transcription of 140 additional genes present in the KT2440 chromosome, including horizontally acquired genes [55]. The extent of the effect produced by the absence of Sfh and Pmr, is likely a result of their structural similarities to the xenogeneic silencers H-NS and MvaT which allow these homologs to oligomerize and bind AT-rich DNA. The Sfh protein has the capacity to form heterodimers with H-NS and its paralog StpA [44,57], and the occupancy of Sfh expands to include 645 of the 745 H-NS-unique target genes in a dominant-negative *hns* background [67]. Likewise, Pmr can interact with other MvaT family proteins present in the KT2440 chromosome, i.e., the TurA and TurB proteins, which possess overlapping binding sites with Pmr [16,55,68]. Less is known about the Acr2 protein compared with Sfh and Pmr. However, recent RNA-Seq experiments carried out with an *Escherichia coli* DH10B strain that carries the pAR060302-derivative plasmid pARΔ*acr2* (modified to eliminate the antibiotic resistance determinants and the Acr2-coding gene) have identified several plasmid and chromosomal genes with altered expression resulting from the *acr2* deletion [56]. Nevertheless, further chromatin affinity purification (ChAP-Seq) assays showed that only three chromosomal genes with altered expression were bound by a 6xHis-tagged Acr2 [56]. It remains to be addressed whether Acr2 can interact with H-NS or StpA. It is worth mentioning that although Dillon et al. [67] observed a complete inclusion of the Sfh-targeted genes within the H-NS regulon, Doyle et al. [60] found that the Δ*sfh* mutation altered the expression of several genes not recognized as regulated by H-NS [69]. The findings of Doyle et al. suggest that Sfh, and perhaps other XS homologs, might be playing regulatory roles independently of H-NS and other XSs.

Lsr2 and Rok homologs have also been identified in plasmids harbored by members of the phylum Actinobacteria and *Bacillus subtilis*, respectively [49,62]. It was found that a short homolog of Rok, encoded by the conjugative plasmid pLS20 of *Bacillus subtilis* strain IFO3335, also serves as a repressor of the master regulator of competence ComK like the full-length Rok encoded by the chromosome [28,62]. Overexpression of Rok_LS20_ in *B. subtilis* strain 168 significantly decreases the transformation efficiency of the strain and reduces gene expression from the *comK* promoter as a result of the Rok_LS20_ binding to this regulatory region. Interestingly, the Rok_LS20_ protein, which corresponds to the C-terminal DNA-binding domain [26], can complement the absence of Rok at least in the competence pathway of *B. subtilis* 168 [62]. Although it was found that Rok_LS20_ likely binds several regions of the *B. subtilis* chromosome [62], experiments addressing the function of Rok_LS20_ in other loci or its interaction with chromosomal Rok are still lacking. Regarding the plasmid-encoded Lsr2 homologs, to the best of our knowledge, there are no published data about their function.

### 3.2. Xenogeneic Silencer Homologs Encoded by Bacteriophages

The increasing number of sequenced bacteriophage genomes has unveiled the presence of phage-encoded homologs of H-NS and Lsr2. Metagenomic analyses of phage-enriched samples from an enhanced biological phosphorus removal (EBPR) bioreactor led to the identification of the EPV1 virus, a parasite of “*Candidatus* Accumulibacter phosphatis” (CAP), which is a member of the community that carries out the EBPR process in that bioreactor [51,61]. The genome of this virus harbors a homolog of the *hns* gene closely related to the *hns* encoded in the CAP chromosome [51]. Although experimental evidence is still lacking, prediction of H-NS binding sites and the identification of low GC regions in the CAP genome led the authors to propose the hypothesis that H-NS_EPV1_ could participate in the modulation of the CRISPR and/or the Type-III restriction-modification systems encoded by CAP, which are known phage-defense systems [51].

Genes encoding homologs of the Lsr2 protein have also been identified in the genomes of the phages Cjw1, 244, Porky, Kostya, and Omega, which infect *Mycobacterium smegmatis* strain mc^2^155 [70,71], and in the CGP3 prophage of *Corynebacterium glutamicum* strain ATCC 13032 [72]. In the latter, ChAP-Seq experiments found that a Strep-tagged version of the CGP3-encoded homolog CgpS bound preferentially to AT-rich regions, mainly at the CGP3 prophage, but also in other chromosomal regions likely acquired by HGT. When a truncated version of CgpS, spanning the N-terminal region, was overexpressed in *C. glutamicum*, derepression of several CGP3-encoded genes and the induction of this prophage was observed [72]. The effect of the truncated version of CGP3 underlines the importance of dimerization/oligomerization in the activity of xenogeneic silencers and their horizontally acquired homologs. A recent review article reported that horizontally acquired Lsr2 homologs are encoded by a great number of other actinobacteriophages sequenced to date, whose hosts belong to genera *Mycobacterium*, *Microbacterium*, *Gordonia*, and *Streptomyces* [63]. As described by the authors of that review, the finding that CgpS is essential to *C. glutamicum* only when CGP3 is present [72], together with the relatively high frequency of Lsr2 homologs (Lsr2_Actinophage_) in lysogenic versus lytic phages, suggests that these XS homologs play a major role in the integration of prophages in bacteria [63]. Nevertheless, CgpS can also bind and modulate the expression of genes outside the CGP3 prophage in *C. glutamicum* [72].

### 3.3. Xenogeneic Silencer Homologs Encoded by Genomic Islands

#### 3.3.1. Genomic Islands

Genomic islands (GIs) are horizontally transferred genetic elements of about 10 to 500 kbp that can integrate into bacterial chromosomes, providing their hosts with advantageous traits [1] such as new metabolic functions, resistance to antibiotics, or virulence factors, among others, which often improve the strain’s overall fitness [1,3,73,74]. Usually characterized by a different GC content, codon usage bias, and dinucleotide frequency relative to their host chromosome [75], GIs are often found at the 3’-end of genes encoding tRNAs and tmRNAs [76]; nevertheless, different GI families may prefer other genes as integration sites [77,78,79]. Under certain conditions, GIs can be excised from the chromosome through the site-specific recombination between the direct repeated sequences (DRSs) that flank the element, in a reaction catalyzed by the integrase protein encoded within or outside the island [80,81,82,83]. This reaction, which is usually promoted by a recombination directionality factor also encoded inside or outside the GI, results in the formation of a circular element [84,85]. The insertion site is reconstituted in the chromosome, and a copy remains as part of the excised GI. These sequences can subsequently take part in the re-integration of the GI into the host chromosome [86]. Besides re-integration, GIs in their circular form can be transferred from one cell to another by means of transduction by co-resident prophages [87] or by conjugation either using their own transfer machinery or taking advantage of the conjugation system encoded by a self-transmissible element [88,89].

Full-length and short homologs belonging to the H-NS family have been identified in genomic islands. While full-length homologs comprise the dimerization/oligomerization and DNA-binding domains of H-NS, the short ones share similarity to one domain only, a feature that provides the latter with anti-H-NS properties that can relieve the H-NS-mediated silencing. Again, similar to what was observed for XS-homologs encoded by plasmids and bacteriophages, the dimerization and DNA-binding capacity of the GI-encoded XSs allow these proteins to regulate the expression of genes located outside their encoding GI.

#### 3.3.2. H-NST, Ler and Hfp (H-NSB)

First reported in 2005, H-NST is a short homolog of the H-NS protein encoded in the so-called *serU* island, a 22.5 kbp pathogenicity island harbored by different strains of pathogenic *E. coli*, such as the uropathogenic (UPEC) strains CFT073 and 536, and also in IE3, a 25.8 kbp genomic island found in the enteropathogenic *E. coli* (EPEC) strain E2348/69 [52]. H-NST corresponds to the dimerization/oligomerization domain of H-NS and behaves as an antagonist of this protein, as observed in experiments where the activity of the H-NS-repressed promoters of *proU* and *bgl* fused to *lacZ,* increased in the presence of a low-level expression of H-NST_EPEC_ [52]. The antagonistic effect is most likely caused by the heterodimers that H-NS and H-NST can form, which would have an altered DNA-binding capacity due to the absence of an H-NS-like DNA-binding domain in the H-NST component [52]. H-NST is also able to increase the expression of genes encoded in the Locus of Enterocyte Effacement (LEE), a genomic island responsible for the attaching/effacing lesions caused by EPEC, enterohemorrhagic *E. coli* (EHEC), *E. albertii*, and *Citrobacter rodentium* [90,91,92,93]. An increase of the amount of the EspA and EspB proteins (exported by the type-III secretion system encoded in LEE) was observed only when H-NST was expressed from a plasmid in the EHEC strain TUV93-0, which lacks the *serU* island, but not when expressed in the EPEC strain E2348/69, which already had high basal expression levels of the LEE-encoded proteins [90]. Whether the high basal expression of the LEE-encoded proteins in EPEC E2348/69 is linked to the presence of the *serU*-encoded H-NST is unknown. Interestingly, it was found through electrophoretic mobility shift assays that H-NST had an intrinsic DNA-binding capacity on two regulatory regions encoded in the LEE island [90]. It is believed that H-NST-binding to these regions might help Ler, another H-NS homolog, to displace the bound H-NS oligomer.

Ler is encoded in the LEE pathogenicity island and, although it has a length comparable to that of H-NS (123 and 137 aa, respectively), the similarities with this XS are restricted to the C-terminal DNA-binding domain [94]. Ler is a master activator of gene expression that works by alleviating the H-NS-mediated repression of the type-III secretion system and its effectors encoded within LEE. Upon binding of Ler to DNA in a non-cooperative manner, a displacement of the bound H-NS takes place [95]. However, the role of Ler is not limited to its genomic island and can activate other virulence factors in pathogenic *E. coli*, such as EspC, which regulates the translocation of effector proteins to host cells and pore formation by EPEC [96], and StcE, encoded in the EHEC O157:H7 virulence plasmid, which contributes to adherence to host cells [97,98]. A similar interplay between Ler and H-NS was observed to modulate the expression from the promoter of the Long Polar Fimbriae in the EHEC strain EDL933, where H-NS represses expression and Ler acts as an antisilencer [99]. Although the formation of dimers and higher-order oligomers of Ler in solution have been observed [54], interaction with H-NS or other xenogeneic silencers has not been reported.

Hfp, also known as H-NSB, is an XS homolog also encoded in the *serU* island, but, unlike H-NST, it is a full-length H-NS homolog [52,53]. In the UPEC strain 536, Hfp was found to play a role in bacterial growth, autoaggregation, hemolytic activity, and specifically in the downregulation of the S and P fimbriae major subunits, the K15 capsule, and expression in the *bgl* operon, which indicates that Hfp acts in a similar fashion as H-NS. Nevertheless, these effects were observed only when the *hfp* mutation was accompanied by an *hns* mutation, suggesting a shared role in the regulation of some genes, which has been supported by the finding of cross-regulation between Hfp and H-NS and their capacity to form heteromeric complexes [53].

#### 3.3.3. The *Enterobacteriaceae*-Associated ROD21-like Genomic Islands

Besides the first report of the occurrence of the H-NS homologs H-NST and H-NSB (Hfp) in the *serU* and IE3 genomic islands made by Williamson and Free in 2005 [52], the assembly of the complete genome sequence of *Salmonella* ser. Enteritidis strain P125109 by Thomson et al. in 2008 revealed the presence of other genomic islands harboring H-NS homologs. The authors identified several regions in the chromosome of the strain P125109 that were absent from the *Salmonella* ser. Typhimurium strain LT2 which were denominated Regions of Difference [100]. Among these horizontally transferred regions, the Region of Difference 21 (ROD21), which includes the genes *SEN1970* to *SEN1999*, was found to encode a putative homolog of H-NS (*SEN1993*). Moreover, the authors showed that other GIs, related to ROD21 and found in *Photorhabdus luminescens* strain TTO1, *Pectobacterium atrosepticum* strain SCR1043, and UPEC strain CFT073, also encoded H-NS homologs.

ROD21 is a 26.5 kpb pathogenicity island inserted in the 3-end of the Asn-tRNA-encoding gene *asnW* of the global *Salmonella* ser. Enteritidis epidemic strains and in the serovars Gallinarum, Dublin, and Nitra [50,100,101]. This GI encodes TlpA, a TIR-domain-containing protein required for the intracellular survival in THP-1 macrophages and the efficient colonization of the murine spleen [102]. Other putative virulence factors likely involved in the colonization of bird and murine internal organs (liver and spleen) are also encoded in ROD21 [103,104]. ROD21 is also an excisable island, a feature that has reached a special relevance due to its role in the virulence of *Salmonella* ser. Enteritidis since different mutant strains, with a reduced excision capacity, show a reduced colonization of the liver and spleen of infected mice [81,105]. The DRSs that flank ROD21 participate in the site-specific recombination reaction that excises the island and produces a circular form of the element [81,106]. The excision process is likely promoted by the products of the genes *SEN1970* and *SEN1998*, which are predicted to encode a tyrosine recombinase and a putative recombination directionality factor, respectively. Indeed, compared with the wildtype strain, the fraction of bacteria with the excised island is reduced in the Δ*SEN1970::FRT* and the Δ*SEN1970::FRT* Δ*SEN1998::FRT* population [50,105,107].

The location of *SEN1970*, downstream of the insertion site in *asnW*, allowed the sequences spanning the corresponding DRS, the *SEN1970* promoter, and the first 82 nucleotides of *SEN1970* to be used to search for genomic islands using BLASTn against the GenBank non-redundant database. This approach identified several genomic islands, phylogenetically related to ROD21, in different species belonging to the family *Enterobacteriaceae* including plant- and animal-pathogenic strains of *Pectobacterium* spp., *Serratia marcescens*, intestinal and extraintestinal *E. coli*, *Enterobacter* sp., carbapenem-resistant *Klebsiella pneumoniae* ST258, and different *Salmonella* serovars, among others [50]. Since ROD21 is the most studied member of this group, it was denominated the *Enterobacteriaceae*-associated ROD21-like (EARL) family of genomic islands. All these GIs share, among other features, the location in an Asn-tRNA-encoding gene and the excision/integration module (the DRSs, and the integrase- and putative RDF-encoding genes), characteristics which allow their excision [50,84,108]. Other genes, such as those encoding putative type-4 pilus-related proteins, relaxases, and type-III restriction-modification systems (type-III R-M), are conserved only in closely related subgroups within the EARL family, likely as a result of their acquisition by an EARL GI followed by the spreading of the island by HGT. The comparative analysis of these GIs also revealed that most of them also have genes encoding full-length and short homologs of the H-NS protein. Indeed, the islands previously reported by Thomson et al. and Williamson and Free as carriers of *hns* homologs, namely HAI7 and HAI13 (*P. atrosepticum* strain SCRI1043) and IE3 (EPEC E2348/69) belong to the EARL family [50,52,100]. Although the *serU* island is also related to ROD21 and encodes H-NSB (Hfp) and H-NST_UPEC_ [52,53], it possesses a different integrase and, therefore, a different integration site. This GI is likely a derivative from IE3, the EARL GI from EPEC E2348/69 which encodes two integrases, the one present in all other EARL islands, and the one present in the *serU* island [52].

**Figure 1:**
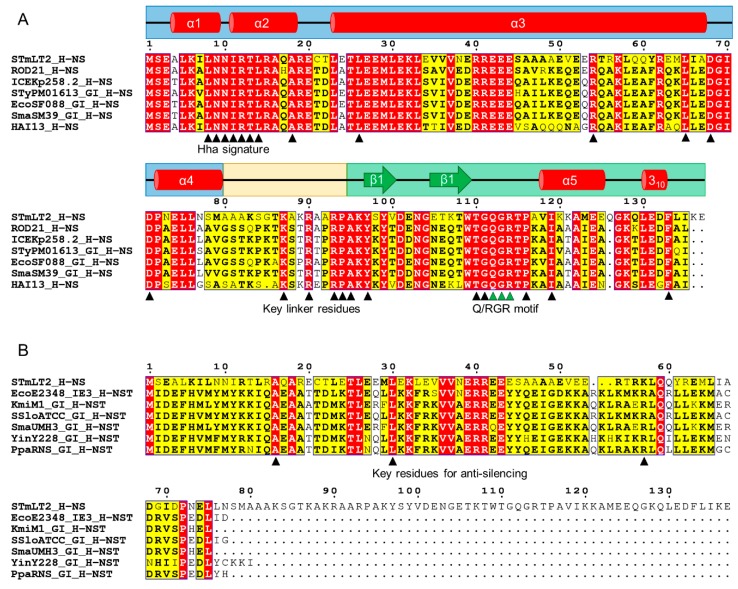
Multiple sequence alignment of the H-NS protein from *Salmonella* ser. Typhimurium LT2 and selected full-length (**A**) and short (**B**) homologs found in EARL genomic islands. The secondary structure elements of H-NS are represented with red cylinders (α-helices) and green arrows (β-strands). The blue, yellow, and green backgrounds represent the dimerization/oligomerization, linker, and DNA-binding domains, respectively. Numbers indicate the corresponding residue of the H-NS protein. The triangles under the alignment indicate the amino acid residues required for function of H-NS (**A**) or H-NST (**B**) that are also conserved in the homologs encoded by the EARL GIs. Aligned proteins correspond to H-NS from *Salmonella* ser. Typhimurium LT2 (STmLT2; WP_001287383.1), full-length homologs encoded in the EARL islands from *Salmonella* ser. Enteritidis P125109 (ROD21; WP_001287371.1), *Klebsiella pneumoniae* ST258 NJST258-2 (ICEKp258.2; WP_001588074.1), *Salmonella* ser. Typhi PM016/13 (StyPM01613_GI; WP_045354302.1), *Escherichia coli* SF-088 (EcoSF088_GI; WP_000005143.1), *Serratia marcescens* SM39 (SmaSM39_GI; WP_041035854.1), *Pectobacterium atrosepticum* SCRI1043 (HAI13; WP_011094424.1), and the short homologs encoded in the EARL islands from *E. coli* O127:H6 E2348/69 (EcoE2348_IE3; WP_000564595.1), *K. michiganensis* M1(KmiM1_GI; WP_038424693.1), *Samonella* ser. Sloterdijk ATCC 15791 (SsloATCC_GI; WP_023201852.1), *S. marcescens* UMH3 (SmaUMH3_GI; WP_089187391.1), *Yersinia intermedia* Y228 (YinY228_GI; WP_042569548.1), and *P. parmentieri* RNS08.42.1A (PpaRNS_GI; WP_033071994.1). The multiple alignment was made in MEGA X [116] using MUSCLE, and the graphic representation was made using ESPript3 [117]. The complete alignment of all 34 full-length and 20 short homologs found in EARL islands is provided as Supplementary Figures S1 and S2.

The EARL GI-encoded full-length and short homologs of H-NS are homologs of H-NSB (Hfp) and H-NST, but represent two different and distantly related clades [50], and, henceforth, they will be denoted as H-NSB_EARL_ and H-NST_EARL_. The full-length homologs share many similarities at the amino acid sequence level with the chromosomal H-NS, including the region interacting with the small nucleoid-associated protein Hha (the Hha signature [109,110,111]), the residues that enters the DNA minor groove for DNA-binding (the Q/RGR motif [35]) and key amino acids at the linker domain also important for DNA-binding [112] (Figure 1A). Since the similarity between H-NS and the H-NSB_EARL_ proteins spans the entire sequence, it is most likely that the secondary and tertiary structures will also be conserved, as suggested by the capacity of the H-NSB protein encoded in the *serU* island to form heterodimers with H-NS and participate in the regulation of known H-NS targets, including virulence factors [53]. Because of the different accessory gene pools present in different species, the regulated or co-regulated genes by the H-NSB_EARL_ proteins are expected to also be different. This represents a subject of further research aimed at better understanding the regulation of virulence in bacterial pathogens. For example, the type-III secretion system 1 (T3SS-1) encoded in the *Salmonella* pathogenicity island 1 (SPI-1) is a key virulence factor that, by translocating effector proteins, allows the invasion of the host cells [113]. Recent findings show that the *Salmonella* ser. Enteritidis Δ*SEN1970::FRT* Δ*SEN1998::FRT* mutant, which have a reduced expression of several ROD21-encoded genes, also have a significantly increased expression of *invA*, a gene encoding a structural component of the T3SS-1 [105]. *invA*, as well as other SPI-1-encoded genes, are upregulated by proteins also encoded by SPI-1 which are, at the same time, negatively regulated by H-NS [114], raising the possibility that the observed link between the two pathogenicity islands could be the result of the H-NSB_ROD21_ protein interacting with H-NS or the H-NS binding sites within SPI-1. As another example, the type-3 pili and the capsular polysaccharide are known H-NS-regulated virulence factors of *K. pneumoniae* [115]. Since the ICEKp258.2 EARL island of the globally spread carbapenem-resistant *K. pneumoniae* ST258 encodes an H-NSB_EARL_ protein (Figure 1A; [50]), this GI-encoded homolog might be playing a role in the virulence of ST258 as well.

Compared with the H-NSB_EARL_ proteins, the short homologs are less related to H-NS, having similarity with the dimerization/oligomerization domain only (Figure 1B), a feature that allows the H-NST_EARL_ proteins to exert a dominant-negative effect that relieves the H-NS-mediated silencing (Section 3.3.2; [52,90]). These proteins have sequence identities ranging from 56.6% to 98.8% and share many conserved amino acids, some of which are important for the activity of the H-NST_EPEC_ protein encoded in the IE3 island from EPEC E2348/69 (Figure 1B). For example, A16, L30, and R60 (positions according to the H-NST_EPEC_ sequence), which are important for the antisilencing and DNA-binding capacity of H-NST_EPEC_ [52,90], are also conserved in the other H-NST_EARL_ proteins (Figure 1B). R63 was shown to be important for the DNA-binding capacity of the IE3-encoded protein [90]; however, in the other H-NST_EARL_ proteins, this position is occupied by different amino acids, the most frequent being K and Q, as previously observed by Levine et al. [90]. It is possible that an H-NS-H-NST interplay, similar to that observed in the LEE island, might be regulating other H-NS-controlled virulence factors in the other enterobacterial pathogens carrying an EARL GI.

**Figure 2:**
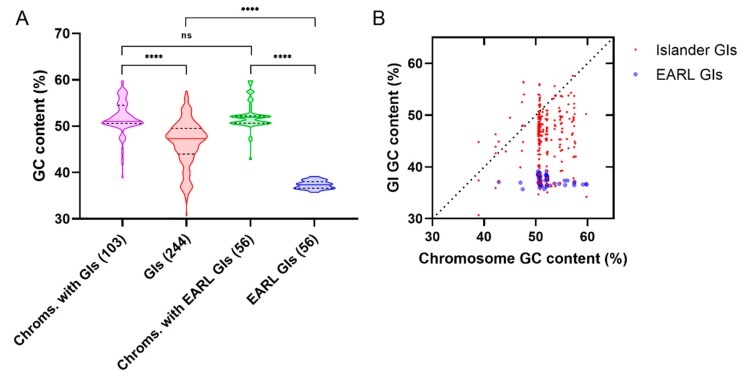
The EARL genomic islands possess a significantly low GC content. (**A**) Violin plots of the GC content of the genomic islands found in *Enterobacteriaceae* in the Islander database (GIs), their host chromosomes (Chroms. with GIs), the EARL genomic islands (EARL GIs) and the chromosomes that harbor the EARL GIs (Chroms. with EARL GIs). Kruskall–Wallis tests followed by Dunn’s multiple comparisons were used to assess differences between medians (α = 0.05, **** *p* < 0.0001). (**B**) Comparison of the GC content of genomic islands and the host chromosome. The dotted line represents a 1:1 correspondence. Data was obtained from the Islander database (15/10/19) [123] and the supplementary information from Piña-Iturbe et al. (2018) [50]. The 597 *Enterobacteriaceae* genomic islands stored in Islander were manually filtered to eliminate possible false positives (indicated in the database), putative prophages (>20% overlap with a PHAST call), duplicated genomic islands (islands with length ≥300 pb in the same species, the same integration site, and GC difference <1%), and islands found in plasmids, resulting in 244 genomic islands present in 103 host chromosomes. All the 56 EARL islands identified in [50] were used.

Genomic islands usually have a lower GC content than the average of their host chromosome [33,80,118,119], a feature also present in the GIs harbored by different species of the family *Enterobacteriaceae* (Figure 2A). Remarkably, the GC content of the EARL islands is significantly lower than the median value of the other *Enterobacteriaceae* GIs (Figure 2A), remaining low even when the host chromosomal GC increases (Figure 2B). This feature agrees with what has been reported for plasmids and actinobacteriophages that encode homologs of H-NS and Lsr2, respectively, which have a lower GC content compared with those which do not encode an XS homolog [63]. The low GC of the EARL islands and its narrow range of variability (35.7–39.2%) could be the result of their relatively rapid spread within *Enterobacteriaceae*, an idea supported by the fact that ROD21 can be transferred by conjugation [106] and that the ICEKp258.2 island from *K. pneumoniae* ST258, which may represent an early member of the SpnT/type-3 R-M-encoding clade within the EARL phylogeny [50], was acquired by this sequence type approximately 20–25 years ago [120,121,122]. Since the XSs show preference for AT-rich DNA, we speculate that these elements have a selective pressure to acquire genes encoding factors that could interact with the silencing effectors from the host cell in order to relieve silencing and provide the opportunity to be incorporated in the host regulatory network. 

#### 3.3.4. Genomic Islands Encode XS Homologs from Different Families 

**Figure 3:**
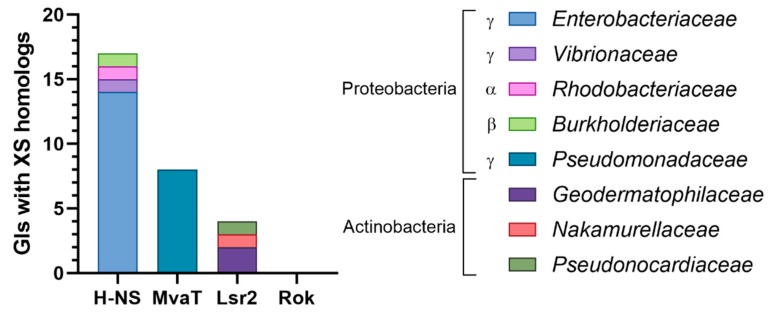
Xenogeneic silencer homologs encoded by genomic islands. The entire Islander database (4065 genomic islands) was interrogated for the presence of xenogeneic silencer homologs using tBLASTn (E-value cutoff = 10) using the amino acid sequences of H-NS (WP_001287383.1), MvaT (WP_003093888.1), Lsr2 (WP_003419513.1), and Rok (WP_003232378.1). The genomic islands corresponding to the BLAST hits were manually filtered to exclude possible false positives, putative prophages, duplicated islands, and islands in plasmids using the same criteria as in Figure 2. Then, the individual hits were manually examined to exclude those corresponding to pseudogenes, alignments outside coding sequences, or alignments in a reading frame different from the annotated coding sequence.

To assess whether GIs encode homologs that belong to the other families of xenogeneic silencers, a tBLASTn search against the entire Islander database of genomic islands (4065 islands) was conducted, followed by manual examination of the resulting hits (Figure 3; Supplementary Table S1). Surprisingly, only 29 genomic islands were found to encode XS homologs, most belonging to the H-NS family, followed by the MvaT and Lsr2 families. No Rok homolog was detected. In agreement with the distribution of the chromosomal XSs among different bacterial taxa (see Section 2.1), the H-NS homologs were found in Alpha, Beta, and Gammaproteobacteria; the MvaT homologs in Gammaproteobacteria (*Pseudomonadaceae*), and the Lsr2 homologs in Actinobacteria. The different numbers of GIs encoding XS homologs can be in part explained by the overrepresentation of the islands from Proteobacteria versus Actinobacteria (64% and 14% of the GIs from bacteria), and *Enterobacteriaceae* versus *Pseudomonadaceae* (55% and 9% of Gammaproteobacteria) in the Islander database [123]. Since *Bacillus* is the only genus in which Rok proteins have been identified, the absence of Rok homologs is likely due to the small number of GIs from *Bacillus* in Islander (46 GIs).

### 3.4. Xenogeneic Silencer Homologs, the Growth Phase and the Environmental Conditions

The observed interaction of the XS homologs with the regulatory networks seems to be more relevant under specific environmental conditions and in different stages of the bacterial life cycle. For instance, in UPEC strain 536, the *hnsB* gene is highly expressed in the stationary phase and at temperatures below 37 °C, while the opposite is observed in the logarithmic phase and 45 °C [53]. Moreover, the effect of an *hnsB* mutation on the generation time, capsule expression, and *bgl* expression is stronger at 25 °C compared with 37 and 42 °C [53]. Contrary to the *hfp* pattern, the expression of *sfh* and *pmr* is high during the log phase, although it was observed that the amount of the Sfh protein increases during the stationary phase and Pmr remains relatively constant along the growth curve [55,124,125]. Further research is required to address the different conditions and contexts that might be modulating the expression of the horizontally acquired XS homologs.

## 4. Concluding Remarks

The main features of the XS proteins (i.e., the preference for AT-rich sequences, a relative lack of binding specificity, and the capacity to form homo and heterooligomers) allow their horizontally acquired homologs to modulate a subset of their regulon through the interaction with the XS proteins and their binding sites, as exemplified by Sfh, Pmr, and Hfp. Although less is known about the other horizontally acquired XS homologs, Rok_LS20_, CgpS and H-NST also modulate the expression of several genes outside the mobile elements that encode these proteins. While most research has focused on the homologs encoded by plasmids and, more recently, by bacteriophages, the XS homologs encoded by GIs have received less attention. Nevertheless, the GI-encoded homolog H-NSB may become of special interest due to its presence in several pathogenic members of the family *Enterobacteriaceae,* including the globally spread carbapenem-resistant *K. pneumoniae* ST258. Moreover, GIs also encode members of the MvaT and Lsr2 families of XSs and, as next-generation sequencing is continuously providing us with more bacterial genomes, additional mobile elements encoding XS homologs will emerge. The current literature shows that the horizontally acquired homologs of XSs play important roles as modulators of gene expression in bacteria, which facilitate horizontal gene transfer, participate in virulence and provide, in some instances, additional growth-phase/environmental-responsive regulation mechanisms.

## Figures and Tables

**Table 1:** Horizontally acquired xenogeneic silencer homologs found in genomic islands, plasmids, and bacteriophages.

XS Homolog	Extent of Similarity (with XS)	Host	Location	Protein–Protein Interaction with ^1^	References
Hfp/H-NSB	full-length (H-NS)	Family *Enterobacteriaceae*	*serU* island and EARL GIs	H-NS	[50,52,53]
H-NST	N-terminal (H-NS)	Family *Enterobacteriaceae*	*serU* island and EARL GIs	H-NS	[50,52]
Ler	C-terminal (H-NS)	Attaching and effacing pathogens (*Enterobacteriaceae*)	Locus of Enterocyte Effacement GI	Ler	[54]
Pmr	full-length (MvaT)	*Pseudomonas putida* strain KT2240	pCAR1 plasmid	Pmr, TurA, TurB, TurE	[55]
Acr2	full-length (H-NS)	Class Gammaproteobacteria	IncA/C plasmids	Unknown	[49,56]
Sfh	full-length (H-NS)	*Shigella flexneri* 2a strain 2457T	pSf-R27 and R27-like plasmids	H-NS, StpA, Sfh	[44,57,58,59,60]
H-NS_EPV1_	full-length (H-NS)	“*Candidatus* Accumulibacter phosphatis”	Phage EPV1	Unknown	[51,61]
Rok_LS20_	C-terminal (Rok)	*Bacillus subtilis* strain IFO3335	pLS20 plasmid	Unknown	[62]
Lsr2 homologs	full-length (Lsr2)	Phylum Actinobacteria	Plasmids (unclassified) and mycobacteriophages	Unknown	[49,63]

^1^ Protein–protein interaction with other chromosomally-encoded XS homologs or the horizontally acquired XS homolog itself.

## Supplementary Materials

The following are available online at https://www.mdpi.com/2073-4425/11/2/142/s1, Figure S1: Multiple sequence alignment of H-NSB_EARL_ proteins, Figure S2: Multiple sequence alignment of H-NST_EARL_ proteins, Table S1: Xenogeneic silencer homologs found in genomic islands of the Islander database.
