# Supplementary figures and images for: Horizontally Acquired Homologs of Xenogeneic Silencers: Modulators of Gene Expression Encoded by Plasmids, Phages and Genomic Islands
Source: Genes (Basel). 2020 Jan 29;11(2):142. doi: 10.3390/genes11020142 (PMC7074111; doi:10.3390/genes11020142)

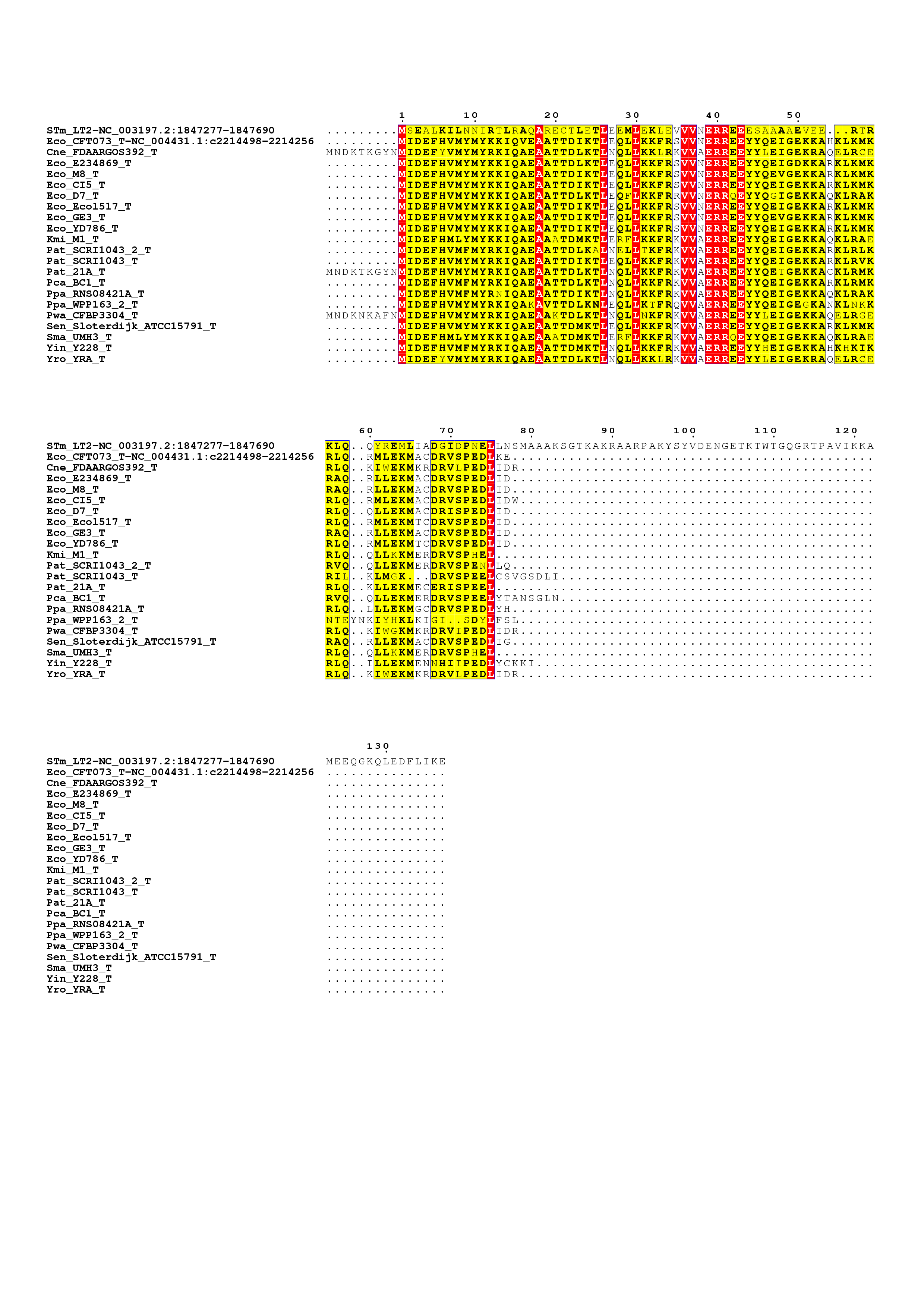

Supplement: Supplementary file 1 [file genes-11-00142-s001.zip › FigS2.tiff]

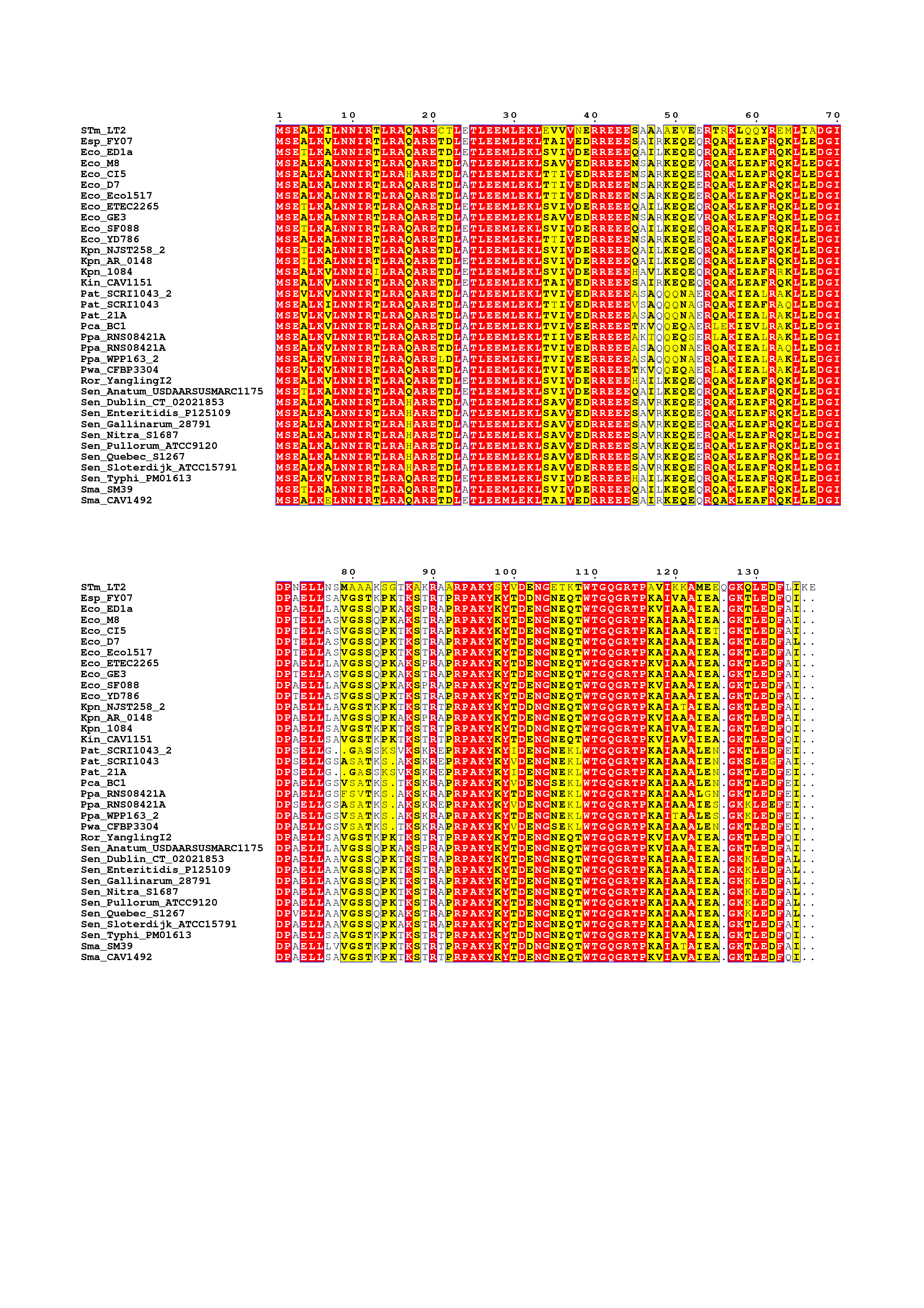

Supplement: Supplementary file 1 [file genes-11-00142-s001.zip › FigS1.tiff]
